# The Autophagic Machinery in Enterovirus Infection

**DOI:** 10.3390/v8020032

**Published:** 2016-01-27

**Authors:** Jeffrey K. F. Lai, I-Ching Sam, Yoke Fun Chan

**Affiliations:** Department of Medical Microbiology, Faculty of Medicine, University Malaya, 50603 Kuala Lumpur, Malaysia; jefferslai@gmail.com (J.K.F.L.); jicsam@ummc.edu.my (I.-C.S.)

**Keywords:** picornavirus, enterovirus, replication, antiviral, autophagy, lipids, autophagosome maturation

## Abstract

The Enterovirus genus of the *Picornaviridae* family comprises many important human pathogens, including polioviruses, rhinovirus, enterovirus A71, and enterovirus D68. They cause a wide variety of diseases, ranging from mild to severe life-threatening diseases. Currently, no effective vaccine is available against enteroviruses except for poliovirus. Enteroviruses subvert the autophagic machinery to benefit their assembly, maturation, and exit from host. Some enteroviruses spread between cells via a process described as autophagosome-mediated exit without lysis (AWOL). The early and late phases of autophagy are regulated through various lipids and their metabolizing enzymes. Some of these lipids and enzymes are specifically regulated by enteroviruses. In the present review, we summarize the current understanding of the regulation of autophagic machinery by enteroviruses, and provide updates on recent developments in this field.

## 1. Enteroviruses

Enteroviruses belong to the family of *Picornaviridae*, and are grouped into 12 species: enteroviruses A to H, enterovirus J, and rhinoviruses A to C. Within these 12 species, many serotypes exist as a result of mutations in the viral structural protein [1]. The serotypes that cause disease in human are formally grouped into five main groups: poliovirus, coxsackievirus, rhinovirus, enterovirus, and echovirus. The icosahedral capsid of enteroviruses consists of 60 copies of four functional subunits, VP1 to VP4. The P1 polyprotein is cleaved to VP0, VP1 and VP3. Subsequently, viral particles mature after cleavage of VP0 into VP2 and VP4. The P2 and P3 polyproteins include the non-structural proteins required for replication. Cleavage of 2A initially occurs, followed by cleavage of the precursor proteins 2BC, 3AB, and 3CD into 2B, 2C, 3A, 3B, 3C and 3D [2].

Enteroviruses cause a diverse range of human diseases, from mild illnesses including undifferentiated fever, conjunctivitis, and hand, foot, and mouth disease, to more severe illnesses such as pneumonia, meningitis, myocarditis, pericarditis, encephalitis, and paralysis [3]. Enterovirus A71 (EV-A71) has been increasingly reported to cause serious illness particularly in young children in Asia [4]. This virus causes hand, foot, and mouth disease, and in rare cases, severe and fatal neurological complications including brain stem encephalitis. In 2014, outbreaks of severe respiratory illness associated with enterovirus D68 occurred among children in the United States [3]. EV-A71 and enterovirus D68 infections are managed only with supportive and symptomatic care, as specific and effective antivirals are not available. Human rhinovirus (HRV) is the main cause of the common cold, and is a significant cause of infective exacerbation of underlying respiratory diseases such as asthma and chronic obstructive pulmonary disease [5]. Effective treatment for HRV is still limited. Coxsackievirus B3 (CV-B3) is an important cause of acute myocarditis, pancreatitis, and meningitis [3].

Poliovirus is the only enterovirus for which an effective vaccine is available. The Global Polio Eradication Initiative, established in 1988, aimed to eradicate polio worldwide by 2000 with the live-attenuated oral polio vaccine [3]. Unfortunately, more than a decade has passed since the targeted date, and the virus remains endemic in a few countries [6]. In view of this, there is also a pressing need to develop novel anti-poliovirus drugs with different mechanisms of action, to be used in the post-vaccine era [7].

The most common strategy in developing antivirals for enteroviruses is to target the viral proteins. However, the high mutation rates of enteroviruses often reduce the effectiveness of these compounds. A switch of target from viral proteins to host factors may be a promising approach, as this would reduce the risk of emergence of enterovirus resistance. The genomic RNA replication of enteroviruses has been shown to occur in membranous vesicles resembling coat protein complex (COPI and COPII)-coated transport vesicles derived from the host secretory pathway during early infection [8,9,10,11]. During late infection, there is a switch to membranes derived from autophagosomes, the degradative compartments of the autophagic machinery, for viral assembly, maturation, and exit from host cells [12,13,14,15]. Therefore, the autophagic machinery could potentially provide novel antiviral targets to treat enterovirus infections. In this review, we summarize the current understanding of the regulation of autophagic machinery by enteroviruses, and provide updates on recent developments in this field.

## 2. The Autophagic Machinery

Autophagy is divided into three distinct processes known as macroautophagy, microautophagy, and chaperone-mediated autophagy. Macroautophagy (hereafter referred to as autophagy) is one of the most widely studied pathways in viruses [16]. Autophagy is a process of homeostasis, and can be activated in response to starvation [17]. Autophagy is also a component of the innate immune response against viral infections [18]. However, some viruses counteract autophagy by expressing proteins that can interfere with the machinery, while other viruses have developed unique mechanisms to thrive in the acidic lysosomal compartments to benefit their replication, spread and survival [19,20].

**Figure 1:**
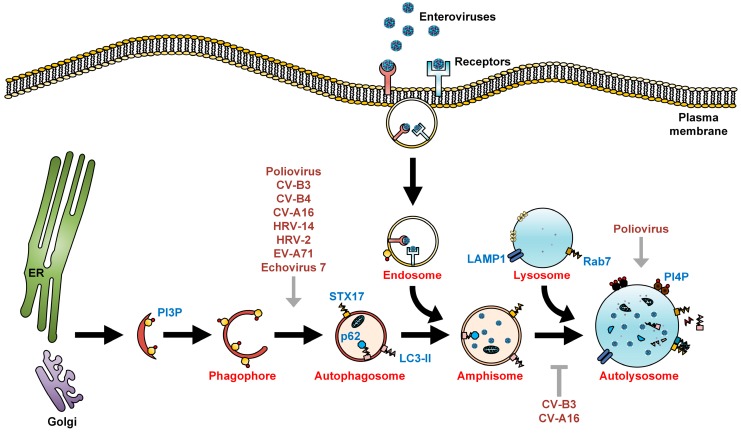
The autophagic machinery and enteroviruses. Autophagy begins with the formation of phagophores that originate from either the endoplasmic reticulum (ER), Golgi, mitochondria, or plasma membrane, or are synthesized *de novo*, and undergo sealing to form autophagosomes. Autophagosomes then undergo a maturation process via fusion with endosomes to form acidic amphisomes. In EV-A71, HRV-A2, HRV-B14, and foot-and-mouth disease virus (FMDV) infections, upon internalization of the virus-receptor complex into endosomes, the acidified endosomes will then trigger viral uncoating [24,25,26,27]. In contrast, poliovirus, echovirus 1, and coxsackievirus A9 do not require endosomal acidification for viral entry [28,29]. During late autophagy, amphisomes fuse with lysosomes to form autolysosomes, where autophagic flux/degradation of sequestered organelles occurs. LC3-II and p62, both markers of autophagic membranes, are also degraded in autolysosomes by lysosomal proteases. Enteroviruses positively (grey arrows) and negatively (grey T arrow) regulate multiple steps of the autophagic machinery for their assembly, maturation, and exit from the host.

Autophagy begins with the generation of phagophores which elongate and self-fuse to form double-membrane vesicles, known as autophagosomes [21]. Autophagy-related genes (ATGs) mediate the formation of phagophores and autophagosomes. The mammalian homolog of yeast ATG8, also known as microtubule-associated protein light chain 3 (LC3), is one well-studied marker of the presence of autophagic membranes [22]. LC3 localizes in the cytoplasm during low autophagic activity, in the form of LC3-I. Upon upregulation of autophagy, LC3-I is conjugated with phosphatidylethanolamine (PE) to form lipidated LC3-PE, also known as LC3-II. The p62 protein interacts with LC3-II to target cargo to the autophagosomes for degradation. Autophagosomes fuse with endosomes to generate amphisomes, which obtain vacuolar-ATPase and become acidic. Subsequently, amphisomes fuse with incoming lysosomes to form autolysosomes. The cargo in the lumina of autolysosomes is then degraded by lysosomal proteases. The p62 protein can also be used to indicate the level of autophagic flux, as this protein is degraded along with LC3-II during complete autophagy (Figure 1) [22,23].

**Figure 2:**
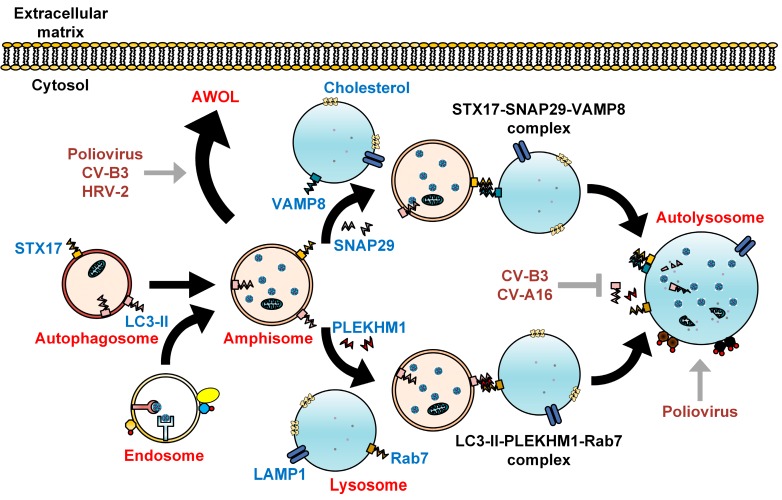
Subversion of autophagosome maturation by enteroviruses. As recently described, autophagosomes fuse with lysosomes via the SNARE complex consisting of STX17, SNAP29, and VAMP8. Alternatively, autophagosome-lysosome fusion can be achieved via LC3-II, PLEKHM1, and Rab7. The engulfed cytoplasmic organelles are then degraded along with the inner membrane of the autolysosome, in a process termed autophagic flux. Cholesterol is commonly found on the membranes of autolysosome. Fusion between autophagosomes and endosomes forms amphisomes bearing viral particles, which can fuse with the plasma membrane to secrete enteroviruses via a proposed process known as autophagosome-mediated exit without lysis (AWOL). Enteroviruses positively (grey arrows) and negatively (grey T arrow) regulate autophagosome maturation to facilitate their own assembly, maturation, and exit from the host.

The late phase of autophagy involves fusion between autophagosomes and lysosomes. The precise mechanism that mediates this fusion event has been recently identified, and involves soluble N-ethylmaleimide-sensitive factor attachment protein receptor (SNARE) complexes, including STX17, SNAP29, and VAMP8 (Figure 2) [30]. Homotypic fusion and protein sorting (HOPS) tethering complex, containing proteins such as vacuolar protein sorting 33A (VPS33A) and VPS16, acts between autophagosomes and lysosomes. These factors are the interacting partners of STX17 [31]. Fusion between autophagosomes and lysosomes is regulated by the adaptor protein SNAP29 via two-way interactions, with SNAP29-STX17 located on the autophagosome membrane and SNAP29-VAMP8 located on the lysosome membrane (Figure 2) [30].

The involvement of Rab7 in autophagosome-lysosome fusion has been well characterized. Rab7 is a small GTPase belonging to the Ras-like GTPase superfamily [32]. In another study, Pleckstrin homology domain containing protein family member 1 (PLEKHM1), an adaptor protein that links LC3 to HOPS and Rab7, was found to mediate maturation of autophagosomes [33]. These findings allow the development of antivirals targeting the STX17-SNAP29-VAMP8 and LC3-II-PLEKHM1-Rab7 complexes.

## 3. Enteroviruses and Autophagy

### 3.1. Poliovirus

**Figure 3:**
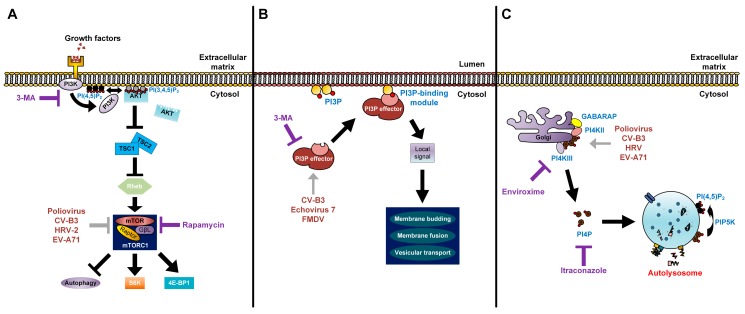
Modes of action of lipids in enterovirus replication. (**A**) The processes of lipid signal transduction regulated by enteroviruses. Class I PI3Ks and their downstream product PI(3,4,5)P_3_ regulate mTORC1 signals. The phosphorylation of PI(4,5)P_2_ via PI3K activates the AKT signaling pathway. AKT blocks the inhibitory effect of TSC1-TSC2 complex on Rheb. Activated Rheb in turn promotes mTORC1 complex signaling. Rapamycin inhibits the mTORC1 complex (violet T arrow) and further enhances production of enteroviruses, which also have an inhibitory effect on mTORC1 (grey T arrow). 3-MA inhibits the class I PI3K and shuts off its downstream signaling (violet T arrow); (**B**) Picornaviruses recruit PI3P effectors (grey arrow) to the outer membranes of autophagosomes. PI3P lipids on autophagosome membranes directly interact with PI3P effectors to induce signals for membrane budding, membrane fusion, and vesicular transport of autophagosomes; 3-MA inhibits the recruitment of PI3P effectors to PI3P lipids (violet T arrow); (**C**) Enteroviruses recruit PI4KIIIβ (grey arrow) via Golgi-localized host factors, resulting in an increase of PI4P lipids. These lipids are commonly found to localize on autolysosome membranes and are converted to PI(4,5)P_2_ by PIP5K for autophagic lysosome reformation. Enviroxime inhibits PI4KIIIβ and itraconazole inhibits cholesterol and PI4P transfers (violet T arrow).

During late infection, the double-membrane vesicles induced by poliovirus have a similar morphology to autophagosomes. These vesicles co-sediment with a variety of markers originating from the ER, Golgi, and lysosomes [34,35,36]. GFP-LC3 constructs have been used to study poliovirus-induced vesicles. These studies reveal co-localization of punctate GFP-LC3 signals with the 3A viral protein, a component of the viral RNA replication complex, and the double-stranded RNA of poliovirus [13,37]. The co-localization between punctate GFP-LC3-expressing cells and lysosomal-associated membrane protein 1 (LAMP1) was also observed, indicating that poliovirus induces autophagosome maturation during infection [37]. Expression of both 2BC and 3A viral proteins were required to induce autophagosome maturation in human embryonic kidney (HEK) 293T cells [37]. Production of intracellular infectious poliovirus in H1-HeLa cells was increased in the presence of autophagy inducers such as rapamycin and tamoxifen (Figure 3A). In contrast, treatment with inhibitors of autophagy, such as 3-methyladenine (3-MA) and RNA interference (RNAi) targeted against LC3 and ATG12, reduced the production of poliovirus (Figure 3B) [37]. Poliovirus also thrives in the mature acidic autophagosomes (Figure 2). Despite the fact that lysosomal proteases are not involved, intracellular vesicle acidification was found to promote the maturation of infectious poliovirus particles [19].

The spread of poliovirus from cell to cell is conventionally thought to require cellular lysis. As a fecal-oral pathogen, the continual release of viral particles into the intestinal lumen without cellular lysis would be beneficial. The escape of poliovirus from the intestine into the central nervous system can lead to destruction of motor neurons, causing acute flaccid paralysis. The finding of cell-to-cell spread of poliovirus in the spinal cords of Bonnet monkeys suggested the possibility of non-lytic spread in the central nervous system. During late infection, neurons in the spinal cord recovered without death of the infected cells [38]. Recently, it has been shown that poliovirus can spread between cells via an autophagy-dependent process known as autophagosome-mediated exit without lysis (AWOL) (Figure 2). The knockdown of LC3 by siRNA reduced the non-lytic spread of poliovirus, while the induction of autophagy with loperamide and nicardipine caused rapid spread of poliovirus in tissue culture and mice [14]. Another recent study demonstrated that clusters of poliovirus particles were sequestered within phosphatidylserine (PS) lipid-enriched autophagosome-like vesicles that are non-lytically released from cells. Interestingly, these vesicles bearing poliovirus had greater infection efficiency compared to free single viral particles [15]. Taken together, these findings indicate that poliovirus usurps the autophagic machinery to promote virus production.

### 3.2. Coxsackieviruses

Coxsackievirus B3 (CV-B3) exploits the autophagic machinery to facilitate its replication on the surface of autophagosomes [39]. The formation of double-membrane vesicles in CV-B3-infected HeLa and HEK293T cells was observed under transmission electron microscopy (EM). The punctate GFP-LC3 and LC3-II/LC3-I ratio were increased upon CV-B3 infection. In the presence of 3-MA and during transient knockdowns of Beclin1, ATG7, and VPS34, the expression of capsid VP1 and CV-B3 viral yield were decreased. In contrast, the treatment of rapamycin increased the expression of VP1 and viral titers (Figure 3A,B). Further investigations revealed that complete autophagy is blocked by CV-B3, as the level of p62 was not affected by viral infection. The inhibition of LAMP2 (the lysosomal transmembrane protein involved in autophagosome-lysosome fusion) increased both VP1 expression and viral production [40]. The results obtained from *in vivo* CV-B3-infected GFP-LC3 transgenic mice were also similar to *in vitro* findings [39,40]. Additionally, large autophagy-related structures bearing RNA polymerase-like components, termed megaphagosomes, were formed in mice infected with CVB3 [40]. Taken together, these findings suggest that CV-B3 subverts the autophagic machinery to facilitate viral replication.

Recently, the silencing of bactericidal/permeability-increasing protein fold-containing family B, member 3 (BPIFB3) was found to enhance the replication of CV-B3 by regulating the induction of autophagy [41]. Receptor interacting protein kinase-3 (RIP3), a regulator of programmed necrosis, increases autophagy flux during CV-B3 infection, which in turn promotes virus replication [42]. Another recent study also found that CV-B3 could replicate using both autophagy-dependent and autophagy-independent pathways *in vivo* [43]. By using three CV-B3 recombinants containing either intact or mutated autophagy-related genes (proLC3, proLC3^G120A^, and ATG4B^C74A^), the infection of CV-B3 was shown to be flexible in the usage of intracellular membranes. The proLC3 virus accumulated large quantities of LC3-II and replicated efficiently via the autophagic pathway. The proLC3^G120A^ protein did not attach to PE to form LC3-II, but in return bound to the ER-resident protein SEL1L, to utilize membranes non-originated from autophagy for CV-B3 replication. The ATG4B^C74A^ protein inhibited LC3-II accumulation, retained LC3-I, and accumulated immature phagophores that were crucial for viral replication [43].

In a recent study, a fluorescent timer protein marker was engineered within a CV-B3 clone to show that CV-B3 was associated with extensive intracellular membrane remodelling and was shed from cells in numerous extracellular microvesicles (EMVs). The detection of LC3-II in these EMVs suggests that the autophagy pathway, likely via the AWOL process, plays an important role in extracellular virus release within microvesicles (Figure 2) [44]. In another recent report, mature CV-B3 was observed engulfed in PS lipid-enriched autophagosome-like organelles and released non-lytically from cells [15].

CV-B4 infection of primary rat neurons has been reported to induce autophagy and accumulation of LC3-II, while 3-MA impaired both the formation of autophagosomes and viral production (Figure 3B) [45]. CV-A16 induces early autophagy, with the 2C and 3C non-structural proteins increasing the accumulation of autophagosomes without promoting fusion between autophagosomes and lysosomes. 2C-induced autophagosome formation was dependent on the human immunity-related GTPase family M protein (IRGM) activation (Figure 1) [46]. However, the role of autophagy in CV-A16 infection and pathogenesis remains unclear.

### 3.3. Human Rhinoviruses

The induction of autophagy has been shown to occur during infections with HRV-B14, a major receptor group virus, and HRV-A2, a minor receptor group virus. The major receptor group uses intracellular adhesion molecule-1 (ICAM-1), while the minor receptor group uses the low-density lipoprotein receptor (LDLR) family for entry. An earlier study demonstrated that HRV-A2 infection was not affected by the autophagy inducer, rapamycin or the autophagic inhibitor, 3-MA [47]. Later, another study showed the co-localization of punctate GFP-LC3 with LAMP1 was evident in both HRV-A2 and HRV-B14-infected cells [48]. Treatment with rapamycin increases HRV-A2 viral titer, while the presence of 3-MA inhibits viral yield, suggesting that autophagy has a role in HRV-A2 replication (Figure 3A,B) [48]. Similar to poliovirus and CV-B3, HRV-A2 is found to be engulfed in autophagosome-like vesicles enriched with PS lipids and released non-lytically into the extracellular matrix (Figure 2) [15].

### 3.4. Enterovirus A71

Autophagy is also activated during EV-A71 infection to facilitate viral replication [49]. Muscle and neuronal cell lines transfected with GFP-LC3 formed GFP+ punctate structures upon infection, and autophagosome-like vesicles were observed in EV-A71-infected cells. The increased accumulation of LC3-II was also evident in virus-infected cells. The viral titer of EV-A71 increased in the presence of rapamycin, but was impaired with 3-MA treatment (Figure 3A,B). Infection of mice with EV-A71 induced double-membrane vesicles in the neurons of the cervical spinal cord. Co-localization of LC3 and VP1 viral proteins was observed in these vesicles using immuno-EM analysis [49].

### 3.5. Echovirus 7

The core components of autophagy are required by echovirus 7 for its entry into host cells via endocytosis. Silencing of genes related to autophagy, including Beclin1, ATG12, ATG14, ATG16, and LC3, as well as 3-MA treatment impaired echovirus 7 infection (Figure 3B) [50]. Taken together, all the studies on poliovirus, coxsackieviruses, rhinoviruses, enterovirus A71, and echovirus 7 showed that utilization of the autophagic machinery is a crucial process for enterovirus infections.

## 4. Regulation of Autophagy by Enteroviruses via Lipids

The early and late phases of autophagy can be governed by lipids and lipid-metabolizing enzymes via three distinct routes. First, they govern the signaling machinery that links to mechanistic target of rapamycin (mTOR), which then regulates the activation of autophagy. This mechanism involves the class I phosphoinositide 3-kinases (PI3Ks) and their product, phosphatidylinositol-3,4,5-triphosphate (PI(3,4,5)P_3_). Secondly, lipids adhere to cytosolic protein mediators to facilitate deformation, expansion, and vesicle transport; an example is the modulation of PI3P that governs the generation and maturation of autophagosomes. Thirdly, the binding of PI4P lipids to autophagosome-lysosome membranes mediates the autophagic machinery [51,52].

### 4.1. The Regulation of Class I PI3K Signaling, PI(3,4,5)P_3_, and mTOR

Homeostasis between growth and starvation is regulated through a nutrient-sensing protein kinase, mTOR. In the presence of nutrients and growth signals, mTOR is activated while inhibiting autophagy (Figure 3A). The inactivation of mTOR during nutrient deprivation slows growth and anabolic processes, stimulating autophagy-mediated degradation of proteins and organelles as a recycling mechanism [53]. The release of growth factors such as insulin simulates the class I PI3K-dependent signaling cascade and activates mTOR complex 1 (mTORC1, Raptor-bound), which in turn suppresses autophagy. The PI(3,4,5)P_3_ converted from PI(4,5)P_2_ via the class I PI3Ks in response to insulin is the initial activator of signaling [53,54]. The phosphoinositide-dependent kinase 1 (PDK1) and AKT proteins are both involved downstream of PI(3,4,5)P_3_ [53,55].

Rapamycin, a potent activator of autophagy, impairs the activation of mTORC1 but not mTORC2 (Rictor-bound), and subsequently inhibits S6 kinase 1 (S6K1) and eIF4E-binding protein 1 (4E-BP1) phosphorylation [53,54]. As mentioned previously, treatment with rapamycin enhances the production of poliovirus, CV-B3, HRV-A2, and EV-A71 infectious progeny [37,39,48,49].

### 4.2. The Control of Class III PI3K Pathway and PI3P

The phosphorylation of PI at the 3’ position of the inositol ring is important for PI3P synthesis via the class III PI3K or VPS34 [55]. The addition of PI3P lipid alone suffices to stimulate the activation of autophagy (Figure 3B). There are two distinct complexes of VPS34 (PI3P effectors), each with its own unique functions [56]. In yeasts, complex I of VPS34, consisting of VPS34, Beclin1, VPS15, and ATG14, localizes at the pre-autophagosomal structure to initiate autophagy. Complex II of VPS34 is similar to complex I, except ATG14 is replaced by UV radiation resistance-associated gene protein (UVRAG), which targets Beclin1 to the endosomes, and functions in vacuolar protein sorting and autophagy induction in mammalian cells. In addition, UVRAG interacts with the class C VPS complex to facilitate fusion between autophagosomes and lysosomes. 3-MA non-specifically inhibits class III and I PI3Ks [57,58,59]. As mentioned previously, the transient knockdown of Beclin1 and VPS34 impairs the expression of VP1 viral protein and CV-B3 viral titers [39]. Echovirus 7 requires Beclin1 and other autophagic components to facilitate its genome release into cytoplasm via the endosomal system [50]. FMDV, a member of the *Picornaviridae* family which is closely related to enteroviruses, requires the interaction of Beclin1 with its 2C non-structural protein to facilitate virus survival (Figure 3B). This interaction is crucial to prevent Beclin1-mediated fusion between autophagosomes and lysosomes. However, the overexpression of Beclin1 in FMDV-infected cells reversed this effect, and facilitated the fusion process [60].

### 4.3. The Roles of PI4P in Autophagy

The yeast PI4-kinase (PI4K) PIK1 (and its vertebrate homolog, PI4KIIIβ) generates PI4P via its effector ATG26 [61]. Under normal physiological conditions, PIK1 and its product PI4P mediate the exit of secretory vesicles from the *trans*-Golgi (Figure 3C). Upon induction of autophagy, the recruitment of ATG proteins from the Golgi networks to phagophores is mediated by PIK1 [62]. The PI4P 5-kinase (PIP5K) facilitates the conversion of PI4P to PI(4,5)P_2_, which is important for autophagic lysosome reformation and recruitment of clathrin to the autolysosomes [63,64]. PI4P and PI(4,5)P_2_ are localized in distinct organelles, with PI4P uniformly distributed on the autolysosomal membrane, while PI(4,5)P_2_ is found on the autolysosomal tubules and buds [64]. Recently, it was reported that γ-aminobutyric acid receptor-associated protein (GABARAP), an autophagy-related protein, recruits PI4KIIα to generate PI4P, and this mediates the fusion of autophagosomes with lysosomes. The PI4P-GABARAP complex is localized along the perinuclear region of Golgi as well as on the autophagosome membrane [65].

In the life cycle of enteroviruses (poliovirus, CV-B3, HRV, and EV-A71), PI4KIIIβ and its product PI4P were identified as host mediators crucial in sustaining the replication of viruses (Figure 3C) [66]. PI4KIIIβ is a downstream effector of ARF1 that catalyzes the synthesis of PI4P lipids in the Golgi membranes [66]. During enterovirus infections, PI4KIIIβ is recruited to the sites of replication enriched with PI4P lipids, which is proposed to attract the viral 3D RNA-dependent RNA polymerase [67]. Apart from 3D polymerase, 2BC of poliovirus interacts directly with PI4KIIIβ. The production of PI4P via PI4KIIIβ and accumulation of unesterified cholesterol through osysterol-binding protein (OSBP) is mediated by 2BC, but is suppressed by 3A and 3AB viral proteins [68]. In contrast, the expression of 2BC precursor protein alone also increased the lipidation of LC3, a characteristic of autophagy induction [69]. Together these findings suggest the connection between PI4P lipids and autophagy as well as the importance of this interplay in the life cycles of enteroviruses.

## 5. Future Perspectives

It is of interest to determine if autophagic degradation of specific targets, including lipophagy (lipids), glycophagy (glycogens), mitophagy (mitochondria), pexophagy (peroxisomes), aggrephagy (protein aggregates), and ER-phagy (endoplasmic reticulum) are exploited by enteroviruses to facilitate their infections [70]. Additionally, the recent identification of specific regulators of autophagosome maturation, including STX17-SNAP29-VAMP8 and LC3-II-PLEKHM1-Rab7 complexes, may enable deeper understanding of mechanisms and roles of autophagy in enteroviral pathogenesis. The non-lytic spread of picornaviruses via exosomes was initially shown during hepatitis A infection, allowing hepatitis A virus to escape from neutralizing antibodies [71]. More recently, enteroviruses were discovered to transmit non-lytically via autophagosome-like vesicles, as populations of viral particles enwrapped in membranes of microvesicles [15]. Vesicular transmission could be beneficial for the overall fitness of enteroviruses, by allowing viral quasispecies to infect susceptible cells [72]. It could provide further insights into different tissue tropism of enteroviruses.

The absence of licensed antivirals against enteroviruses is partly due to high mutation rates of enteroviruses, with a resulting likelihood of emergence of resistance. Knowledge of the role of autophagy in enteroviral infection could lead to a promising area of therapeutics. However, targeting autophagic processes involved in infection may lead to side effects due to the importance of autophagy in cell homeostasis. Therefore, targeting the viral proteins which aid viral infection by interacting with autophagy proteins should provide more selective antiviral action [73]. In addition, lipids obtained from PI3P and PI4P are imperative for the formation of membranous vesicles involved in viral replication. Drugs targeting lipid signaling have shown potential as antivirals against enteroviruses. These include enviroxime, a potent and specific inhibitor of PI4KIIIβ, and itraconazole, an inhibitor of OSBP that shuts off cholesterol and PI4P transfers (Figure 3C) [74,75]. Recent advances in computational drug design have led to the identification of compounds that target the PI4KIIIβ lipid kinase and have potent antiviral activity against HRV, poliovirus, coxsackievirus, and EV-A71 [76]. However, the potential autophagy-modifying effects of these drugs have not yet been confirmed or correlated with their antiviral effects, which would be an area of considerable interest for future work.

## 6. Conclusions

In conclusion, the expanding knowledge of the relationship between enteroviruses and the autophagy pathway points to fascinating, diverse and complex mechanisms that can positively or negatively impact the survival of both viruses and host. Unraveling these mechanisms will provide insights into enterovirus life cycles and host immune responses, and a potential source of future drug development.
